# A Fracture Liaison Service to Address Vitamin D Deficiency for Patients Hospitalized for Osteoporotic Fracture

**DOI:** 10.1210/jendso/bvae050

**Published:** 2024-03-12

**Authors:** Xiaoxu Sun, Benjamin Z Leder, Marcy B Bolster, Thuan V Ly, Esteban Franco-Garcia, Charles T Pu, WuQiang Fan

**Affiliations:** Department of Endocrinology and Metabolism, Shanghai Tenth People’s Hospital, Tongji University School of Medicine, Shanghai, 200072, China; Division of Endocrine, Massachusetts General Hospital, Harvard Medical School, Boston, MA 02114, USA; Division of Endocrine, Massachusetts General Hospital, Harvard Medical School, Boston, MA 02114, USA; Division of Rheumatology, Massachusetts General Hospital, Harvard Medical School, Boston, MA 02114, USA; Harvard Orthopaedic Trauma Initiative, Massachusetts General Hospital, Harvard Medical School, Boston, MA 02114, USA; Division of Geriatrics, Massachusetts General Hospital, Harvard Medical School, Boston, MA 02114, USA; Division of Geriatrics, Massachusetts General Hospital, Harvard Medical School, Boston, MA 02114, USA; Division of Endocrine, Massachusetts General Hospital, Harvard Medical School, Boston, MA 02114, USA

**Keywords:** osteoporotic fractures, treatment gap, vitamin D deficiency, inpatient, fracture liaison service

## Abstract

**Context:**

Addressing vitamin D deficiency (VDD) is important for fracture secondary prevention.

**Objectives:**

To explore the function of a fracture liaison service (FLS) to address VDD.

**Design, Setting and Patients:**

An observational study of patients admitted to the Massachusetts General Hospital with fractures between January 1, 2016, and October 31, 2023, cared for by the FLS.

**Intervention:**

Ergocalciferol 50 000 international units (50ku-D2) oral daily for 3 to 7 days.

**Main Outcomes Measures:**

VDD prevalence. Efficacy of inpatient daily 50ku-D2 in raising serum 25-hydroxyvitamin D (25OHD) levels.

**Results:**

Of the 2951 consecutive patients, 724 (24.53%) had VDD (defined by 25OHD ≤ 19 ng/mL). Men (252/897, or 28.09%) were more likely than women (472/2054, or 22.98%) to have VDD (*P* = .003). VDD was seen in 41.79% (117/280), 24.41% (332/1360), and 20.98% (275/1311) of patients of aged ≤59, 60 to 79, and ≥80 years, respectively (*P* < .00001). Of the 1303 patients with hip fractures, 327 (25.09%) had VDD, which was associated with a longer length of stay (8.37 ± 7.35 vs 7.23 ± 4.78 days, *P* = .009) and higher trend of 30-day-readmission rate (13.63% vs 18.35%, *P* = .037). In a cohort of 32 patients with complete data, each dose of 50ku-D2 increased serum 25OHD by 3.62 ± 2.35 ng/mL without affecting serum calcium or creatinine levels.

**Conclusion:**

VDD was seen in nearly 25% of Massachusetts General Hospital FLS patients and more prevalent in male and younger patients. VDD was associated with longer length of stay and higher 30-day-readmission risk in patients with hip fracture. Daily 50ku-D2 appeared to be a practical way to quickly replete vitamin D in the inpatient setting.

Vitamin D is a pleiotropic hormone that has receptors and biological effects in a variety of tissues [1]. The hormone plays an important role in skeletal health and may facilitate bone mineralization and enhance bone mineral density by increasing intestinal calcium and phosphorus absorption, reducing secondary hyperparathyroidism, and decreasing bone turnover [2].

Although the ability of vitamin D administration alone to reduce fracture risk remains unclear [3-5], there is less controversy that vitamin D has significant implications for bone health care in individuals who have already experienced a fragility fracture because of underlying osteoporosis [2, 6] and that addressing vitamin D deficiency (VDD) is an important component of secondary fracture prevention [1, 7].

The optimal level of vitamin D remains ill-defined and is believed to be condition/disease dependent. For skeletal health, the Institute of Medicine [8] and the Endocrine Society [9] recommended dietary and supplemental allowances to achieve a total serum 25-hydroxyvitamin D (25OHD) level of at least 20 ng/mL. Patients with a serum 25OHD level of less than 20 ng/mL are generally considered to have VDD.

VDD is common among patients admitted to the hospital with fragility fractures, including hip fractures [10-12]. VDD is associated with negative rehabilitation outcomes, reduced ambulation after surgery[13], increased fall risk [1], impaired fracture healing[14], and orthopedic material osseointegration [7]. It is also generally held that vitamin D synergizes with osteoporosis treatment [15].

A fracture liaison service (FLS) has emerged as an effective care model to improve osteoporosis treatment rates, prevent future fractures, and reduce the fracture-associated morbidity, mortality, and health care costs [16-18]. An FLS also offers a potentially effective mechanism to address VDD in patients with a recent fragility fracture.

Here, we report real-world data on vitamin D status among consecutive patients hospitalized at Massachusetts General Hospital (MGH) with osteoporotic fractures who were cared for by the MGH FLS. VDD prevalence, its associated clinical outcomes, and strategies to address VDD in the acute postfracture inpatient setting were studied.

## Subjects and Methods

The study was performed at MGH and approved by the institutional review board of Mass General Brigham (MGB).

Patients admitted to the MGH for a fragility fracture between January 1, 2016, and October 31, 2023, and cared for by the MGH FLS were included in this observational study.

The clinical data from the MGB health care system database of Research Patient Data Registry, a centralized clinical data registry that has gathered more than 5 billion clinical observational facts on the entire MGB patient population, was applied for the present study.

For all patients, data on demographic characteristics, laboratory results (including 25OHD, serum creatinine, serum calcium), dates of inpatient admission or emergency department visit, operative date, discharge date, diagnosis of nonunion or malunion, inpatient medication administration records specifically those for ergocalciferol, cholecalciferol, and calcium were obtained from the Research Patient Data Registry. The data are valid up to October 31, 2023.

All patients cared for by the MGH FLS receive a comprehensive bone health evaluation, which includes laboratory assay for 25OHD level as part of the initial laboratory tests on admission [16]. The vitamin D assay used at MGH is a delayed 1-step competitive immunoassay using the chemiluminescent microparticle immunoassay technology and has an inter- and intra-assay coefficient of variance of 4.5% and 3.6%, respectively. The assay does not differentiate free from vitamin D-binding protein (VDBP)-bound form, nor does it differentiate D2 from D3.

VDD is defined as a 25OHD level of 19 ng/mL or less. To address VDD in the acute postfracture inpatient setting, the MGH FLS adopts a vitamin D repletion regimen consisting of ergocalciferol 50 000 IU oral daily for 3 to 7 days, with a mean of 4 days followed by maintenance cholecalciferol of 1000 to 1500 IU daily. This regimen is started within 0 to 1 days after the initial 25OHD result. For a subset of patients who received daily ergocalciferol, a repeat 25OHD levels were also obtained 1 to 3 days after the last dose of ergocalciferol. Patients with 25OHD level >20 ng/mL were treated with cholecalciferol 1000 to 1500 IU daily. Because the effect on the fracture risk reduction of vitamin D in combination with calcium is more consistent compared with that of vitamin D alone [19-21], all patients under MGH FLS care, regardless of 25OHD level, receive 650 to 1000 mg per day of elemental calcium with very rare exception [22].

Statistical analysis was performed using IBM SPSS Statistics version 26, R version 3.5. and GraphPad Prism version 9.5. Discrete variables are compared using the chi-squared test and continuous variables by *t* tests. The readmission rates were assessed with the use of Kaplan-Meier curves, with the last date of data collection (October 31, 2023) used as a censor. The significance of between-group differences in incidence was calculated with the use of a log-rank test. Cox proportional hazard regression analysis was used to estimate the hazard ratio and 95% CI. A *P* value less than .05 was considered to be statistically significant.

## Results

### VDD Among FLS Patients

Of the 2951 consecutive patients admitted to the MGH with fractures between January 1, 2016, and October 31, 2023, who were cared for by the FLS and with valid 25OHD levels, with the latter assessed at the time of admission, 724 (24.53%) were vitamin D deficient as defined by 25OHD level of 19 ng/mL or less. VDD was seen in 28.09% (252 of 897) of male patients, which was higher than that of 22.98% (472 of 2054) in female patients (*P* = .003). VDD was seen in 41.79% (117 of 280), 24.41% (332 of 1360), and 20.98% (275 of 1311) of patients of aged 59 years or younger, 60 to 79 years, and 80 years or older, respectively (*P* < .00001). These data are summarized in Table 1.

**Table 1.** VDD among 2951 consecutive fracture patients cared for by the MGH FLS between January 1, 2016, and October 31, 2023

Group	N total	N of patients with VDD	%	*P*
Total	2951	724	24.53	N/A
Age (y)-specific subgroups				
≤59	280	117	41.79	<.00001
60-79	1360	332	24.41	
≥80	1311	275	20.98	
Sex-specific subgroups				
Female	2054	472	22.98	.003
Male	897	252	28.09	

Abbreviations: FLS, fracture liaison service; MGH, Massachusetts General Hospital; N/A, not available; VDD, vitamin D deficiency.

### VDD Among Patients With hip Fracture

Table 2 shows that VDD was detected in 327 of 1303 (25.10%) patients with hip fragility fractures. In the hip fracture population, similar to the overall FLS population, younger age was associated with a higher prevalence of VDD, such that VDD was seen in 36.21% (21 of 58), 27.36% (151 of 552), and 22.37% (155 of 693) of patients aged 59 years or younger, 60 to 79 years, and 80 years or older, respectively (*P* = .02).

**Table 2.** VDD among the 1303 patients with hip fractures

Group	N total	N of patients with VDD	%	*P*
Total	1303	327	25.10	N/A
Age (y)-specific subgroups				
≤59	58	21	36.21	.02
60-79	552	151	27.36	
≥80	693	155	22.37	
Sex-specific subgroups				
Female	889	214	24.07	.21
Male	414	113	27.29	

Abbreviations: N/A, not available; VDD, vitamin D deficiency.

Hip fracture patients with VDD, despite being younger (78.55 ± 11.75 vs 80.28 ± 10.47, *P* = .018), had longer length of stay (LOS) than those without VDD (8.37 ± 7.35 vs 7.23 ± 4.78 days, *P* = .009). The extra day of LOS was mainly the result of prolonged postsurgical LOS (6.88 ± 7.06 vs 5.61 ± 4.57 days, *P* = .003) (Table 3).

**Table 3.** LOS of the entire hospitalization or that since surgical date of the 1303 hip fracture patients according vitamin D status

Group (n)	Age (mean ± SD)	M/F ratio	LOS total (days)	LOS since surgery (days)
VDD (n = 327)	78.55 ± 11.75	113/214	8.37 ± 7.35	6.88 ± 7.06
Non-VDD (n = 976)	80.28 ± 10.47	301/675	7.23 ± 4.78	5.61 ± 4.57
*P*	.018	.21	.009	.003

Abbreviations: F, female; LOS, length of stay; M, male; VDD, vitamin D deficiency.

**Figure 1.**
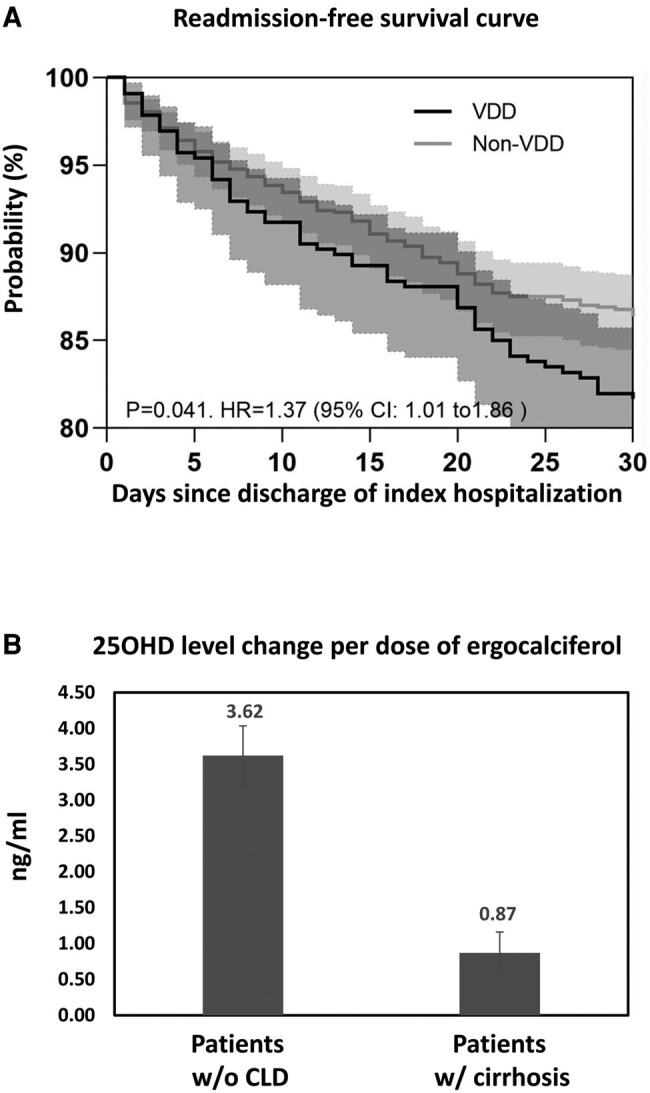
(A) Kaplan-Meier analysis of the cumulative rates of readmission during the initial 30 days following the index hip fracture hospitalization among patients who were vitamin D deficient at initial admission, and those who were not. Error bars (shaded areas) represent 95% CIs. Hazard Ratios (HR) and *P* value of a log-rank test are also shown. (B) Changes of serum 25 hydroxyvitamin D (25OHD) levels per dose of 50 000 IU ergocalciferol in patients with and without cirrhosis. Shown are mean ± standard error, *P* = .002. W/, with; W/O, without.

The cumulative rates of readmission, defined as visit(s) to emergency department or inpatient admission for any reason, was analyzed during the 6 months following the discharge day of the index hip fracture hospitalization. Compared with those with 25OHD level of ≥20 ng/mL, patients with VDD had higher risk of readmission during the initial month (Table 4). The difference dissipated by 6 months post index fracture hospitalization. A Kaplan-Meier survival analysis (Fig. 1A) showed a higher readmission rate by 30 days in patients with VDD (hazard ratio, 1.37; 95% CI, 1.01-1.86; *P* = .041).

**Table 4.** Readmission rate of patients with hip fractures according vitamin D status

Group (n)	Readmission (n, %) by number of days since discharge		
	30	60	180
VDD (n = 327)	60 (18.35%)	82 (25.08%)	117 (35.78%)
Non-VDD (n = 976)	133 (13.63%)	199 (20.39%)	303 (31.05%)
*P* (of χ^2^)	.037	.074	.113

Abbreviations: VDD, vitamin D deficiency.

### VDD and Risk of Fracture nonunion

Rates of nonunion diagnosis in the 2-year durations preceding and after the index fracture according to admission level of 25OHD level were then analyzed. A diagnosis of nonunion within 90 days following the index fracture is considered as existing rather than new onset of nonunion. As shown in Table 5, when the entire FLS population was assessed, 3.31% of patients with VDD at index fracture hospitalization had a diagnosis of nonunion in the preceding 2 years, the corresponding rate for that of the patients without VDD was 3.55%, and the difference between the groups were not significant. The rates of new diagnosis of nonunion (1.24%) during the 2 years following the index fracture in the VDD group did not differ significantly when compared with the non-VDD group (1.03%). A similar pattern was seen among patients whose index hospitalization was for hip fracture.

**Table 5.** Nonunion diagnosis rates during the 2 years preceding and the 2 years following the index fracture according to vitamin D status

Groups	Non(mal)-union From –730 days to 90 days*^a^*			Non(mal)-union From 91 days to 730 days		
	n	Rate (%)	*P*	n	Rate (%)	*P*
All FLS patients (n = 2951)						
VDD (n = 724)	24	3.31	.77	9	1.24	.64
Non-VDD (n = 2227)	79	3.55		23	1.03	
Patients with fractures (n = 1303)						
VDD (n = 327)	11	3.36	.65	4	1.22	.89
Non-VDD (n = 976)	28	2.87		11	1.13	

Abbreviations: FLS, fracture liaison service; VDD, vitamin D deficiency.

^*a*^A diagnosis of nonunion within 90 days following the index fracture is considered as existing rather than new onset of nonunion.

### A Course of Daily Ergocalciferol for VDD

Among 32 patients, without known malabsorption or liver cirrhosis, who had 25OHD levels available before and after the daily ergocalciferol-based vitamin D repletion regimen, each dose of ergocalciferol, on average, raised serum 25OHD level by 3.62 ± 2.35 (mean ± SD) ng/mL.

Serum calcium levels before and after the ergocalciferol course were 8.73 ± 0.64 and 8.85 ± 0.65, respectively (*P* = .91). Similarly, the ergocalciferol course was not associated with changes in serum creatinine levels (1.01 ± 0.62 before and 0.97 ± 0.79 after, *P* = .96).

### VDD in Fracture Patients With Chronic Liver Disease

We then studied VDD prevalence in patients with chronic liver disease (CLD), with the latter being defined as cirrhosis, viral hepatitis, autoimmune hepatitis, metabolic dysfunction-associated fatty liver disease, alcoholic liver disease, primary biliary cholangitis, and other rare etiologies such Wilson disease. VDD was seen in 64 of 156 (41.04%) patients with CLD; the prevalence is significantly higher than that of 23.58% (659 of 2795) in patients without CLD (*P* < .00001). Among 10 patients with cirrhosis with 25OHD levels available before and after a course of daily ergocalciferol treatment, each dose of ergocalciferol raised serum 25OHD by 0.87 ± 0.92 (mean ± SD), which was significantly lower than that of patients without cirrhosis (3.62 ± 2.35 ng/mL; *P* = 0.002; Fig. 1B).

## Discussion

The present study explored the function of an FLS, in real-world practice of a single institution, to address VDD among patients admitted with fragility fractures.

The MGH FLS was established in 2016, with the primary goals of improving the osteoporosis care rate and secondary fracture prevention among patients who were admitted with fragility fracture [16]. As part of a comprehensive bone health care approach, the MGH FLS has a multifaceted role in addressing VDD through systematic screening at admission, patient counseling and education, prompt repletion/supplementation, ongoing monitoring of vitamin D status, as well as communication and collaboration with longitudinal care providers.

Our data show that VDD is seen in nearly 25% of patients admitted with fragility fractures including hip fractures, a prevalence lower than that previously reported in the literature where up to 40% to 80% of patients with fracture were VDD [12, 13, 23-25]. The prevalence of VDD of our study patient cohort is however similar to that of the general US population (24.6%) according to National Health and Nutrition Examination Survey (2001-2018) data [26]. Our data suggest that younger age is associated with higher prevalence of VDD, which is also consistent with the National Health and Nutrition Examination Survey data[26]. The reasons for this age differences, though not well defined, could include higher rate of vitamin D supplementation [27] and lower rate of sunscreen use [28] among older people. Of interest, the rates of preadmission vitamin D prescription in our study cohort were higher in younger than in older patients (data not shown), which we believe might reflect health care providers’ intervention to higher VDD prevalence seen in younger patients.

For patients admitted with hip fractures, we observed that a 25OHD level ≤19 ng/mL was associated with, on average, 1 day longer of hospitalization, primarily from a prolonged postsurgical LOS. Although this finding is in line with what's reported by Lim et al [29], association between vitamin D status and LOS after hip fracture appears inconsistent between studies [30]. Of note, low vitamin D levels showed an inverse association to the LOS among patients underwent elective arthroplastic surgery [31].

The available data on the effects of vitamin D on human fracture healing remain inconclusive [32]. Although a large cohort study of 309 330 human fractures did find that VDD is associated with a slightly increased nonunion rate (odds ratio, 1.14 [1.05-1.22]) [14], the data of the current study show that the nonunion rate of patients with VDD did not differ from that of patients with 25OHD of ≥20 ng/dL. Nevertheless, it remains possible that our study has insufficient power to detect a possible difference given the infrequency of nonunion. Of note, a relatively small randomized controlled trial involving 102 patients (mean age, 29 years) who received an intramedullary nail for a tibia or femoral shaft fracture showed that high-dose vitamin D supplementation at the time of fracture was not associated with difference in clinical or radiographic healing at 3 months and >12 months [33].

An additional area of interest is whether VDD impacts readmission rates following a hip fracture hospitalization. Our study results suggest that VDD is associated with higher readmission rate during the initial month, but not for longer duration, a finding that is generally in agreement with results reported by Ingstad et al [34].

In adults, a dose of 50 000 IU of ergocalciferol once a week for 8 weeks is often effective in correcting VDD [6, 9], and is currently a common way of treating VDD [9]. This regimen works well for the general ambulatory patient population who is able to have regular outpatient follow up. Patients with recent fragility fractures, who are often elderly, frail, and poly-morbid, could however experience significant difficulty in achieving timely and regular outpatient follow-up [10, 16]. It is for this reason that the MGH FLS adopts a regimen of daily ergocalciferol of 50 000 IU for 3 to 7 days followed by maintenance dose of cholecalciferol to promptly replete vitamin D for fractures patients with VDD during the index fracture hospitalization. Our data suggest that in the acute postfracture and inpatient setting, each dose of 50 000 IU of ergocalciferol raises serum 25OHD level by 3 to 4 ng/mL, without affecting serum calcium or creatinine levels, among patients with no known gastrointestinal malabsorption or liver cirrhosis. We believe this inpatient daily ergocalciferol regimen offers an effective and safe mechanism to address VDD for this particularly at-risk population. Of note, available literature suggests that dose-response of serum 25OHD level to vitamin D supplementation is nonlineal and is with individual variations. Baseline level of serum 25OHD is among multiple factors that contribute to the individual variation. Patients with lower baseline 25OHD levels were reported to have a more robust response to vitamin D replacement [35, 36]. The 25OHD response to ergocalciferol reported in this study was observed in patients with baseline 25OHD level of <20 ng/mL and may not be readily applicable to patients without VDD.

Although one of the main purposes and anticipated benefits of addressing VDD is to minimize the risk of hypocalcemia associated with potent antiresorptives such as zoledronic acid [10, 22], the potential clinical outcome of our inpatient vitamin D repletion regimen (eg, its effect on improving osteoporosis pharmacotherapy rate) remains to be evaluated.

It is worth emphasizing that the present vitamin D repletion regimen was administered with careful monitoring in an inpatient setting and has specific target patient population which is postfracture patients who are often elderly and frail and are at risk of “failing” a transition to daily cholecalciferol from conventional weekly ergocalciferol dosing regimen. There are reports suggesting that higher dose vitamin D replacement in older adults is associated with increased risk of fall and hospitalization in either community-dwelling [36, 37] or institutionalized patients [38]. Long-term high-dose (≥4000 IU) daily cholecalciferol in otherwise healthy adults is associated with reduced volumetric bone mineral density and increased risk for hypercalciuria [39]. Furthermore, vitamin D supplementation might also be associated with impaired muscle health [40].

Our FLS practice has identified fracture patients with CLD as a particularly challenging population in terms of addressing VDD. Compared with the general fracture population, patients with CLD have a higher (41%) prevalence of VDD, and among them, those with cirrhosis demonstrated a significantly blunted response to ergocalciferol repletion as shown by diminished 25OHD elevation after each dose of ergocalciferol. Of note, >75% of patients with CLD, regardless of underlying etiology, develop osteoporosis hence are at an elevated fracture risk [41, 42]. The underlying pathophysiology of this condition, now more commonly termed hepatic osteodystrophy, remains unclear and represents an emerging area of active research [43-46]. Optimal treatment for hepatic osteodystrophy is unknown, though regular supplementation of vitamin D and calcium is believed to be a cornerstone of bone therapy. The mechanism of the blunted response to ergocalciferol repletion is uncertain and is likely multifactorial, potentially involving impaired gastrointestinal absorption from gut microbiota change [47] or severe cholestasis or intestinal mucosa edema associated with portal hypertension; it may also involve impaired VDBP synthesis by the cirrhotic liver, as well as impaired hepatic 25-hydroxylation (CYP2R1 activity) [46]. Future studies are clearly warranted to explore an effective vitamin repletion strategy in patients with CLD and VDD.

Like most other hospitals, the MGH pathology lab measures total 25OHD level, and assay for VDBP is not routinely available for clinical care purpose. Lack of VDBP levels is one of the limitations of the present study because the total 25OHD levels could be interfered with by fluctuations/variations of VDBP levels, and these fluctuations/variations could occur in the settings of acute illness such as bone fracture, differential sex hormonal levels, and hepatic synthetic functional status. It would also be of interest to measure levels of 24,25-dihydroxyvitamin D [24,25(OH)2D] because it is suggested that the vitamin D metabolite ratio [24,25(OH)2D/25OHD] is independent of VDBP levels and may provide a superior index of vitamin D status [48]. 24,25(OH)2D is also reported to be required for optimal repair of bone fractures [49]; it would therefore be of interest to investigate whether this metabolite is associated with LOS and/or readmission rates.

In conclusion, nearly 25% of patients hospitalized at MGH for osteoporotic fractures and seen in consultation by the MGH FLS had VDD. Younger age, male sex, and CLD were associated with a higher prevalence of VDD. VDD was associated with, on average, 1 extra day of hospital stay and higher readmission rate during the initial month among patients admitted for hip fracture. Daily ergocalciferol, with each dose of 50 000 IU raising serum 25OHD level by 3 to 4 ng/mL, followed by maintenance dose of cholecalciferol of 1000 to 1500 IU is potentially a practical way to quickly replete vitamin D among patients with VDD hospitalized for fracture.

## Data Availability

Some or all datasets generated during and/or analyzed during the current study are not publicly available but are available from the corresponding author on reasonable request.

## Abbreviations

24,25(OH)2D: 24,25-dihydroxyvitamin D
25OHD: 25 hydroxyvitamin D
CLD: chronic liver disease
FLS: fracture liaison service
LOS: length of stay
MGB: Mass General Brigham
MGH: Massachusetts General Hospital
VDBP: vitamin D-binding protein
VDD: vitamin D deficiency
